# Thermo-Responsive Hydrogel Containing Microfluidic Chitosan Nanoparticles Loaded with *Opuntia ficus-indica* Extract for Periodontitis Treatment

**DOI:** 10.3390/ijms25179374

**Published:** 2024-08-29

**Authors:** Raffaele Conte, Anna Valentino, Ilenia De Luca, Gemilson Soares Pontes, Anna Calarco, Pierfrancesco Cerruti

**Affiliations:** 1 Research Institute on Terrestrial Ecosystems (IRET)-CNR, Via Pietro Castellino 111, 80131 Naples, Italy; anna.valentino@cnr.it (A.V.); ilenia.deluca@cnr.it (I.D.L.); 2 National Biodiversity Future Center (NBFC), 90133 Palermo, Italy; 3 Laboratory of Virology and Immunology, National Institute of Amazonian Research (INPA), Manaus 69067-375, AM, Brazil; gemilson.pontes@inpa.gov.br; 4 Post-Graduate Program in Basic and Applied Immunology, Institute of Biological Science, Federal University of Amazonas, Manaus 69077-000, AM, Brazil; 5 Institute for Polymers, Composites, and Biomaterials (IPCB-CNR), Via Campi Flegrei 34, 80078 Pozzuoli, Italy; pierfrancesco.cerruti@cnr.it

**Keywords:** thermo-responsive hydrogel, *Opuntia*, microfluidic particle synthesis, periodontitis, anti-biofilm, macrophage modulation

## Abstract

Periodontitis is a chronic inflammatory disease resulting from the dysbiosis of periodontal bacteria and the host’s immune response, leading to tissue degradation and sustained inflammation. Traditional treatments, such as mechanical debridement and antimicrobial agents, often fail to fully eradicate pathogenic bacteria, especially in deep periodontal pockets. Consequently, the need for novel therapeutic approaches has increased the interest in bioactive natural extracts, such as that of *Opuntia ficus-indica*, known for its anti-inflammatory, antioxidant, and antimicrobial properties. This study investigates the encapsulation of *Opuntia ficus-indica* extract in OFI-loaded chitosan nanoparticles (OFI-NPs) via ionotropic gelation using a microfluidic system, allowing precise control over nanoparticle characteristics and enhancing protection against enzymatic degradation. To achieve localized and sustained release in periodontal pockets, a thermo-responsive hydrogel comprising hyaluronic acid and Pluronic F127 (OFI@tgels) was developed. The transition of OFI@tgels from a solution at low temperatures to a solid at body temperature enables prolonged drug release at inflammation sites. The in vitro application of the optimized formulation eradicated biofilms of *S. mutans*, *P. aeruginosa* (PAO1), and *P. gingivalis* over 36 h and disrupted extracellular polymeric substance formation. Additionally, OFI@tgel modulated immune responses by inhibiting M1 macrophage polarization and promoting a shift to the M2 phenotype. These findings suggest that OFI@tgel is a promising alternative treatment for periodontitis, effectively reducing biofilm formation and modulating the immune response.

## 1. Introduction

Periodontitis is a chronic, multifactorial inflammatory condition caused by biofilm that results from the complex interaction between periodontal bacteria and their by-products. This interaction stimulates the host’s inflammatory response, leading to ongoing inflammation and tissue degradation. The tooth-associated biofilm undergoes dysbiosis with a progressive transition from Gram-positive aerobic bacteria to Gram-negative and anaerobic species, such *Porphyromonas gingivalis*, *Tannerella forsythia*, and *Treponema denticola* (called “red complex” bacteria). This transition affects the gingival environment in terms of pH and oxygen levels, favoring species that thrive in this habitat [1]. Furthermore, the imbalance in microbial biofilm and the host immune response induces neutrophils and macrophages to continuously produce proinflammatory mediators, such as growth factors, cytokines, matrix metalloproteinases (MMPs), and reactive oxygen species (ROS). The abundance of inflammatory mediators in periodontitis promotes the growth of specific bacteria that thrive on pro-inflammatory agents. This, in turn, creates a self-perpetuating cycle of inflammation and increases the presence of periodontal pathogens that drive and sustain this disease [2,3]. Thus, restoring homeostasis to regain the complex anatomical periodontium structure is the goal for periodontal treatment. A typical periodontitis remedy involves mechanical debridement with the remotion of supra- and subgingival deposits from root surfaces and the application of antimicrobial agents. Nevertheless, this approach has a limited likelihood of success and is influenced by factors such as individual adherence, smoking habits, tooth position, and the severity of the disease. Chlorhexidine and systemically administered antibiotics are sometimes used in conjunction with the physical removal of biofilms. However, in some instances, these treatments fail to completely eradicate pathogenic bacteria, especially those that penetrate deep periodontal pockets or invade periodontal tissues [4]. In addition, topical formulations, such as mouthwashes, rinse systems, and agents containing iodine, do not produce the desired outcomes when used over an extended period. Therefore, it is necessary to develop new therapeutic approaches to address the limitations of the current treatments [5,6].

In recent years, the use of bioactive natural extracts has increased significantly in the management of periodontitis due to their multiple pharmacological activities [7]. In this context, *Opuntia ficus-indica*, also known as the prickly pear or nopal cactus, has been used in traditional folk medicine for centuries owing to its richness in bioactive compounds, such as polyphenols, betalains, and vitamins, which possess anti-inflammatory, antioxidant, and antimicrobial properties [8]. There is various evidence that *Opuntia ficus-indica* flowers, seeds, and fruit, along with their extracts, are active both in vitro and in vivo against Gram-positive and Gram-negative bacteria [9,10]. However, the utilization of OFI extract as a liquid formulation is not helpful, as it quickly retains biological fluid and loses its rheological characteristics [9].

The encapsulation of OFI extract may serve as an effective alternative approach to maintain its biological activity, facilitating regulated release and prolonging its presence within the periodontal pocket.

This contribution aimed to synthesize a thermo-sensitive injectable hydrogel (OFI@tgel) based on hyaluronic acid (HA) and Pluronic F127 (F127) containing OFI-loaded chitosan nanoparticles (OFI-NPs) as a local drug delivery system into periodontal pockets with antibacterial and antioxidant properties.

This hydrogel allows in situ triggered and reversible transitions between the solution phase (low temperature) and solid state (body temperature) in the periodontal pocket, maintaining stable and sustained drug release at periodontal lesions.

OFI-loaded chitosan nanoparticles (OFI-NPs) were prepared by ionotropic gelation in a microfluidic system using sodium tripolyphosphate (TPP) as anionic crosslinking agents and chitosan (CS) as structural polymer [11]. The microfluidic approach represents a valid alternative to conventional bulk methods for achieving precise and reproducible nanoparticle (NP) production. This technique allows for accurate control of key factors influencing drug delivery systems’ performance, such as particle size, size distribution, and polydispersity index (PDI), through precise adjustments in reagent concentrations and fine-tuning of the flow ratio. This method effectively safeguards polyphenols against enzymatic oxidation or degradation. The optimized formulation was able to eradicate in vitro *S. mutans*, *PAO1*, and *P. gingivalis* biofilm over 36 h, interfering with extracellular polymeric substance (EPS) formation. In addition, OFI@tgel affects the expression level of several virulence genes involved in cell-to-cell communication, seriously hampering biofilm formation/maturation. OFI@tgel inhibited the polarization of the M1 macrophage phenotype while concurrently facilitating the transition to the M2 phenotype.

## 2. Results and Discussion

### 2.1. OFI Extract

*Opuntia ficus-indica* (OFI) prickly pear by-products, including peel, pulp, and seed, represent a beneficial source of dietary fiber, bioactive molecules, vitamins, as well as minerals (calcium, magnesium, potassium, and manganese) that exhibit noteworthy biological activity, including anti-inflammatory, hypoglycemic, and antibacterial properties [12,13,14]. Bellumori et al. [15] summarized the mineral, polyphenol, and pigment composition of discarded OFI fruits from two Sicilian locations compared to fresh commercial prickly pear samples. Analysis of prickly pear by-products, including peel, pulp, and seed, revealed substantial mineral content, particularly potassium, calcium, and magnesium, with peel samples showing the highest concentrations. Flavonoids emerged as the predominant phenolic class in both peel and whole fruit. Statistical analysis indicated no significant differences in mineral and total phenolic content between discarded and commercial fruits. Similarly, Scarano et al. characterized high-value by-products from OFI Miller fruit peel using traditional (i.e., maceration) and advanced (i.e., Naviglio Extractor^®^) extraction techniques [16]. Ethanol and ethanol-water extracting solutions demonstrated higher total phenolic compound content, with significant differences observed between extraction methods.

UHPLC-ESI-MSn analyses performed by Mena and colleagues on prickly pears from six Spanish cultivars [17] identified up to 41 phenolic compounds, including ferulic acid-hexoside, dihydrosinapic acid-hexoside, and sinapic acid-hexoside. Phenolic composition varied significantly depending on the part of the plant. Similarly, other researchers have observed a similar pattern for phenolic compounds, particularly flavonoids, in fruits from Mexican and Spanish cultivars [18,19]. A consistent qualitative pattern in both early- and late-maturation fruits was found, with negligible differences in the concentration of the primary polyphenols across the yellow (Sulfarina), white (Muscaredda), and red (Sanguigna) cultivars of *Opuntia* spp. In the present study, the quantitative characterization of the phenol compounds present in OFI was based on retention time, fragmentation patterns, and comparison with the available literature (Table S1). The findings of the present study, as detailed in Table 1, corroborated existing literature and demonstrated that the OFI hydroethanolic extracts were rich in isorhamnetin, quercetin, kaempferol, ferulic acid, and their derivatives (kaempferol-3-O-glucoside, quercetin-3-O hexose deoxyhexose, narcissin or isorhamnetin rutinoside, kaempferol-3-O-hexose deohyhexose, and rutin).

The observed qualitative and quantitative differences in OFI phytochemical profile depend on various factors such as geographical origin, genetic variety, climate, harvest season, storage conditions, and processing [20].

### 2.2. Preparation and Physicochemical Characterization of OFI-Loaded Nanoparticles (OFI-NPs)

To optimize the microfluidic process of chitosan nanoparticle preparation, a T-shaped polydimethylsiloxane device for microfluidic was selected, having two lateral input channels for the solution of sodium tripolyphosphate (TPP), one central channel for chitosan solution, and one releasing conduit for the fabricated NPs (Figure S1). This platform effectively controls the mixing, separation, and interaction of fluids at the intersection [21,22]. Manufacturing parameters (middle flow speed, side flow speed, flow rate ratio (FRR), chitosan concentration, and TPP concentration) were optimized to ensure nanoparticle monodispersity [23,24,25]. As detailed in Table S2, the smallest NPs (92.0 ± 13.0 nm) were achieved with a chitosan/TPP FRR of 0.143. Higher FRR values led to an increase in nanoparticle size, likely due to material accumulation inside the microchannels and the subsequent formation of microfiber-like structures [26]. As shown in Table S3, NPs with the best morphology were obtained using 0.3% (*w*/*v*) chitosan in acetic acid (0.1 M) with TPP (0.15 mg/mL) at an FRR of 0.143 (50 µL/min of the middle stream and 350 µL/min side flows). The optimized parameters were subsequently utilized for the synthesis of OFI-loaded nanoparticles (OFI-NPx, where x refers to the OFI extract concentration dissolved in the TPP solution). The addition of varying amounts of dried OFI extract significantly affected the nanoparticles’ polydispersion index (PDI) and encapsulation efficiency. At the lowest OFI concentrations (0.1–0.4 mg/mL), size and PDI remained below 100 nm and <0.2, respectively, but the encapsulation efficiency was low. Increasing the OFI concentration above 0.6 mg/mL, the size distribution curve shifted to larger values, ranging from 135.0 ± 21.0 nm in OFI-NP_0.8_ to 223.0 ± 27.0 nm in OFI-NP_1.2_ with a higher PDI. Moreover, the incorporation of OFI into NPs led to a significant (*p* < 0.05) decrease in zeta potential values, from 32.4 ± 0.5 mV in OFI-NP_0.1_ to 12.3 ± 1.63 mV in OFI-NP_1.2_, indicating weakened physical stability (Table S4). Similar trends have been observed in other studies where herbal extracts were encapsulated into chitosan NPs [27,28]. This can be ascribed to the potential discharge of OFI extract from the surface of nanoparticles, which can cause NP aggregation.

**Figure 1:**
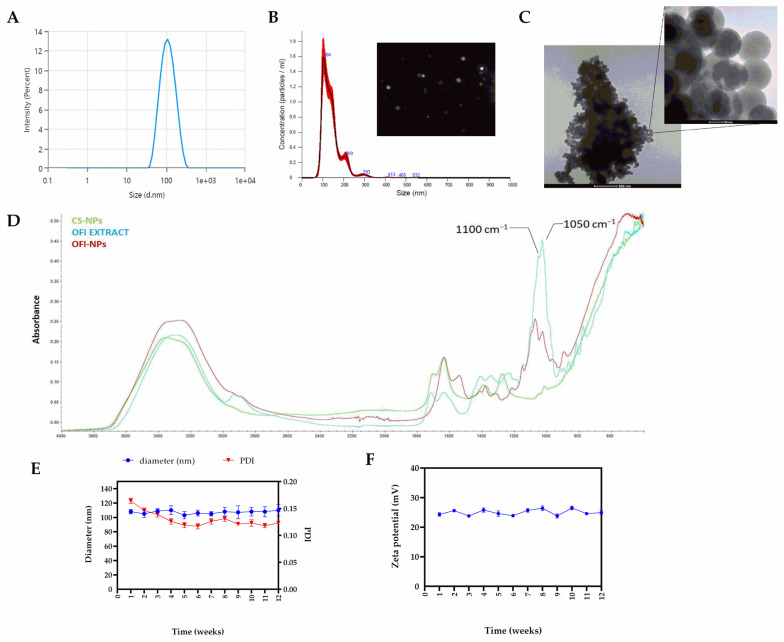
Physicochemical characterization of optimized OFI-loaded nanoparticles (OFI-NP_s_). (**A**) Average particle size, and (**B**) NTA measurement of OFI-loaded nanoparticles in suspension. The frame is a representative screenshot of the NTA video. (**C**) Morphology of OFI-loaded nanoparticles using TEM microscopy. (**D**) FTIR spectra of OFI-NPs. (**E**) Size, PDI, and (**F**) ζ-potential of OFI-NPs during three months of storage at 25 °C.

The physicochemical properties of OFI-loaded nanoparticles after optimization are shown in Figure 1. OFI-NP_0.6_, hereafter referred also to as OFI-NPs, evidenced a monomodal population with an average diameter of 100.0 ± 13.1 nm (Figure 1A), a polydispersity index (PDI) < 0.2, and a ζ-potential of 30.9 ± 0.5 mV. A similar size distribution was obtained by NTA analysis (Figure 1B). Moreover, high-resolution transmission electron microscopy showed that OFI-NPs were spherical in shape, with an average diameter of about 85.2 ± 5.4 nm. The difference between the TEM value and DLS results might be attributed to the dry state of the nanoparticle during TEM measurement [29]. Successful loading of OFI was confirmed using FTIR analysis through the characteristic absorption peaks at 1050 cm^−1^ and 1100 cm^−1^, suggestive of the –C-O stretching and –OH deformation vibrations of alcohols derived from the extract [30] (Figure 1D). Furthermore, NP stability analysis at 25 °C showed no significant changes in size, PDI (Figure 1E), and ζ-potential (Figure 1F) over three months of storage.

### 2.3. Preparation and Characterization of the F127-Based Formulations Containing OFI-NPs

Injectable hydrogels have gained significant interest as a highly promising treatment in periodontitis due to their exceptional attributes, including porosity for the in situ release of bioactive molecules, fluid absorbency, viscoelastic properties for adhering to various tissues, biocompatibility, and the ability to customize their properties for diverse applications. Moreover, their programmable sol–gel responsiveness under specific stimuli minimizes trauma during use, allowing for tailored formulations to improve treatment efficacy, enhancing patient comfort and compliance, making them versatile tools in periodontal care [31,32].

Pluronic F127 (F127) is an FDA-approved thermo-responsive polymer commonly used for smart drug delivery formulations, such as nasal, ophthalmic, and vaginal [33]. However, in highly diluted water solutions, F127 quickly loses its capacity to gel. Previous work has demonstrated that the addition of high-molecular-weight hyaluronic acid (HA, Mw: ~1000 kDa) to F127 increases the mechanical strength of the resulting hydrogel without affecting its free-flowing ability and thermosensitive property [34].

**Figure 2:**
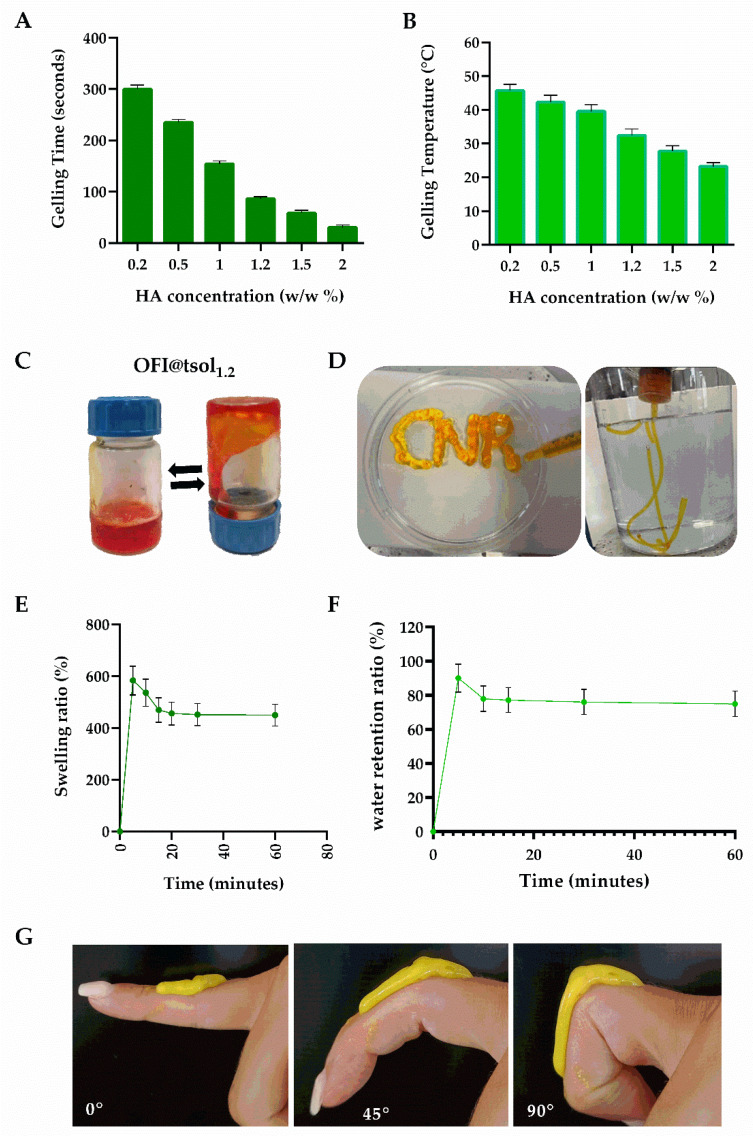
OFI@tsol and OFI@tgel properties. Gelling time at 33 °C (**A**), and temperature (**B**) of OFI@tsol. Appearance of OFI@tsol and OFI@tgel (**C**,**D**). OFI@tgel swelling ratio (**E**); water retention ratio of OFI@tgel at 35 °C (**F**). Adhesion properties of OFI@tgel (**G**).

Hence, to improve the targeted delivery of OFI extract on gingival inflamed tissues, a series of OFI-loaded F127-based sols (OFI@tsol_x_) were prepared by mixing dried OFI-NPs (10 mg) with cold F127 (20 wt%) and different amounts of HA (0.2–2 wt%). As expected, the presence of HA did not hamper the thermally triggered F127 process, which leads to the formation of a gel (OFI@tgel_x_), while affecting both gelation time and temperature. As HA concentration increased, the gelation time at 33 °C decreased from 300.4 ± 8.2 s for OFI@tgel_0.2_ to 30.1 ± 2.1 s for OFI@tgel_2_ (Figure 2A). Similarly, the lowest gelation temperature was observed around 23.2 ± 0.4 °C when HA concentration was 2 wt%, while the sol containing 1.2 wt% HA (OFI@tsol_1.2_) showed a sol-to-gel transition at 32.4 ± 0.3 °C (Figure 2B), a value similar to the oral mean temperature of 36.5 °C [35]. OFI@tsol_1.2_ was a syringeable solution at 25 °C, transforming its state into a gel at a temperature around 33 °C (Figure 2C,D). According to these results, OFI@tgel_1.2_ (further referred to as OFI@tgel) was considered for subsequent investigations.

An ideal hydrogel for periodontitis treatment should have the dual capability to absorb excess exudate from gum lesions while maintaining adequate moisture to prevent the treatment area from drying out [36]. The water absorption and retention properties of OFI@tgel were determined by the weighing method. As shown in Figure 2E, OFI@tgel swells rapidly within the first 5 min of immersion in simulated salivary fluid (SSF) at 35 °C, reaching the swelling equilibrium after 20 min of immersion. Moreover, the water retention of the hydrogel gradually decreases, losing the excess absorbed water after 1 h (Figure 2F). Another relevant property of a hydrogel for periodontitis treatment is its adhesion to oral tissues. Zhou et al. described several PDA-modified hydrogels with suitable adherence to oral tissues given by optimized gel water retention abilities [37]. Similarly, Pham et al. developed thermosensitive hydrogels by blending 28% *w*/*v* Pluronic F127 with varying concentrations of methylcellulose (MC) and silk fibroin (SF). These hydrogels were designed for the localized delivery of metronidazole to oral infection sites, utilizing their swelling properties for controlled release [38,39]. Therefore, a qualitative test to assess the adhesion properties of OFI@tgel to soft tissue was performed by extruding the OFI@tsol on a finger through a 25-gauge syringe. As shown in Figure 2G, the extruded sol maintained its shape after injection due to the sol-to-gel transition and was able to firmly adhere to soft tissue after adapting to the finger’s movements (Figure 2G). Overall, injectability, elasticity, and tissue adhesion features allow OFI@tgel to effectively fill irregular tissue defects through straightforward injections, leading to its application in the gum task.

### 2.4. OFI Release and Antibacterial Activity of OFI@tgel

**Figure 3:**
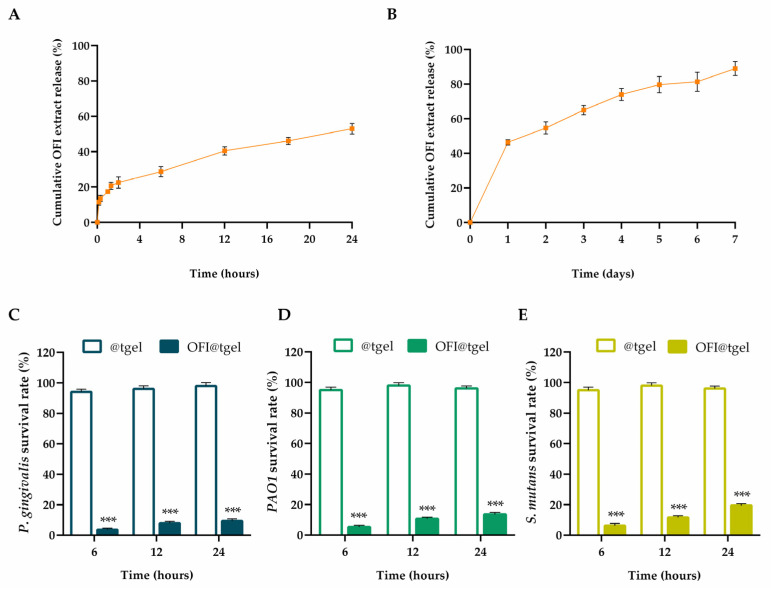
OFI extract release and antibacterial activity. Cumulative OFI release from OFI@tgel simulated salivary fluid (SSF) at pH 6.5 and 35 °C after 24 h (**A**) and 1 week (**B**). Antibacterial activity of OFI@tgel relative to *P. gingivalis* (**C**), *PAO1* (**D**), and *S. mutans* (**E**) as survival rate (%) at 6, 12, and 24 h. The results are expressed as the means of the values obtained (mean ± SD) after six measurements for each sample. Statistically significant differences: *** *p* < 0.001 versus @tgel.

Free radicals are abundant at the inflamed gum site, causing excessive oxidative stress and metabolic disorders. To ensure that the antioxidant activity of the OFI extract persists over time, the release kinetics and the antioxidant capability of OFI released from OFI@tgel were evaluated (Figure S2). Samples were placed on a porous glass set with the hydrogel lower surface in contact with simulated salivary fluid (SSF) at pH 6.5 and 35 °C. As reported in Figure 3A, the OFI release kinetic is characterized by modest burst release during the first hour (~18%), followed by a gradual and sustained release during 1 day of incubation. OFI release depends on a two-phase release process: during the first few hours, the extract diffuses from the nanoparticle surface to the hydrogel matrix before being released into the gingival tissue. Over time, swelling of the hydrogel increases the leakage of OFI-NPs, resulting in a further increase of OFI concentration in the SSF (Figure 3B).

Periodontitis and periodontal diseases are infectious processes of the oral cavity, resulting from a delicate balance between microbial challenge and the host’s immune response. Disruption of this equilibrium leads to the clinical manifestations of the disease. Several studies have reported the antibacterial properties of *Opuntia ficus-indica* extract. Roya Pourmajed et al. demonstrated that ethyl acetate and ethanolic extracts of OFI shows antibacterial activity against *E. coli* recovered from patients with urinary tract infection [40]. Dhaouadi and colleagues observed effective antimicrobial activity, particularly against *Staphylococcus aureus* and *Staphylococcus epidermidis* of the traditionally made Tunisian OFI syrup with a minimal bactericide concentration (MBC) of 1.3 mg phenolics/mL [41]. In another study, the OFI skin fruit extracts exhibited significant (*p* < 0.01) antibacterial activity against *S. typhi*, *B. subtilis*, and *S. pneumoniae* than tetracycline and vancomycin [42]. *Porphyromonas gingivalis*, *Pseudomonas aeruginosa* (*PAO1*), and *Streptococcus mutans* are among the most harmful periodontopathogens isolated from the periodontal pockets of patients with chronic periodontitis [43,44]. Thus, these strains are widely used as standard pathogenic bacteria for studying the efficacy of compounds against oral pathogens. As shown in Figure 3C–E, all tested pathogens were extremely susceptible to OFI@tgel, with the inhibitory rates ranging from 90.7% for *Porphyromonas gingivalis* to 80.5% for *Streptococcus mutans* after 24 h.

### 2.5. OFI@tgel Antibiofilm Activities

The management of periodontitis continues to be a significant clinical challenge due to the complex interaction between dental biofilm microorganisms and the host’s inflammatory response, which drives a degenerative process in the surrounding tissues [39]. Because of the host’s immune defense mechanisms and tolerance to antimicrobial therapy (e.g., systemic antibiotic, antifungal therapy, or antiseptic topical), periodontal infections with mono- or poly-microbial biofilms are common and difficult to treat [45]. Indeed, biofilm provides an inimitable environment able to facilitate bacterial cell-to-cell communication through the production of signaling molecules, which promote collaborative behavior. Simultaneously, stochastic processes and non-uniform gene expression can result in the emergence of bacterial subpopulations with distinct morphologies and environmental reactions [46]. In addition, mature biofilms release planktonic bacteria to infect the nearby tissues and induce chronic inflammation [47].

**Figure 4:**
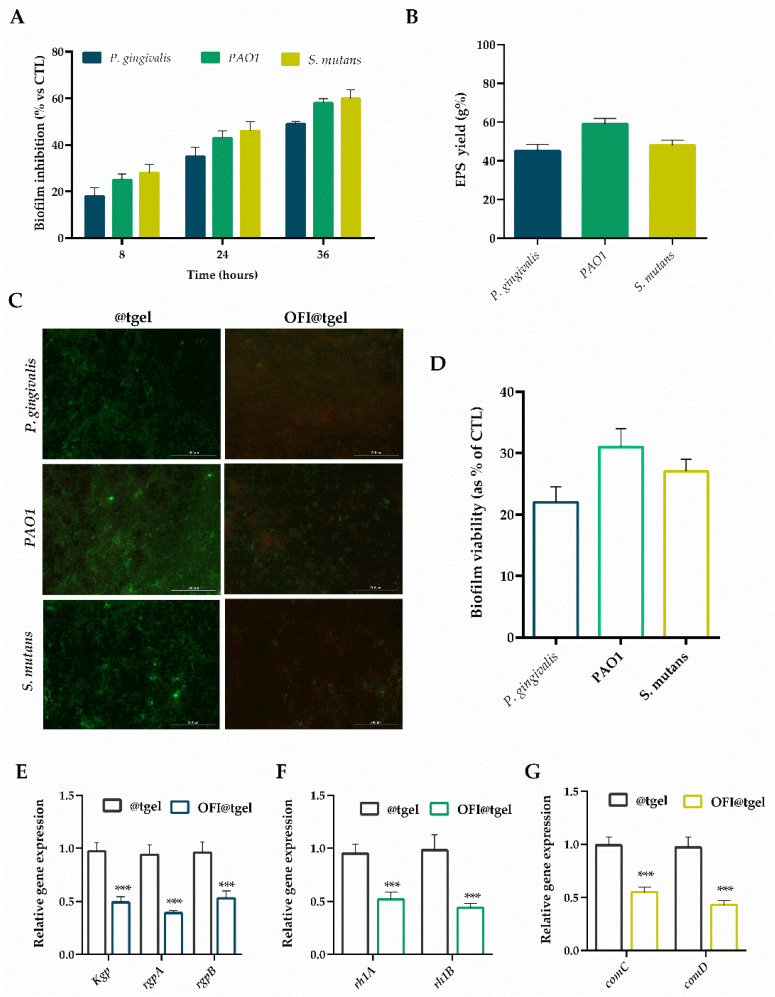
Antibiofilm activity of OFI@tgel. (**A**) *P. gingivalis*, *PAO1*, and *S. mutans* biofilm inhibition was evaluated by crystal violet after 8, 24, and 36 h of incubation at 35 °C in the presence of OFI@tgel. (**B**) EPS production in the presence of OFI@tgel in *P. gingivalis*, *PAO1*, and *S. mutans* compared to hydrogel alone. (**C**) Fluorescence microscopy images of live/dead staining (scale bar 200 µm, 40× magnification). Live bacteria appeared green, while dead bacteria were stained red. When live and dead bacteria were in close proximity, they produced a yellow/orange color. (**D**) Quantification of biofilm viability with or without OFI@tgel. mRNA expression level of QS-related genes after incubation with OFI@tgel in (**E**) *P. gingivalis*, (**F**) *PAO1*, and (**G**) *S. mutans*. The expression levels of the tested genes were normalized to the mean critical threshold (CT) values of the housekeeping gene 16s rRNA using the 2^−ΔΔCt^ method. Hydrogel without OFI extract was used as a control (@tgel). For each sample, six independent experiments were performed, and the results were expressed as mean ± SD. *** *p* < 0.001 compared to @tgel.

Therefore, the capability of OFI@tgel in biofilm eradication was examined on *S. mutans*, *PAO1*, and *P. gingivalis* strains over 36 h. As shown in Figure 4A, OFI@tgel exhibited remarkable efficacy in thwarting the initial development of biofilms, with an efficacy of about 82% for *P. gingivalis*, 75% for *PAO1*, and 72% for *S. mutans* after 8 h. Moreover, OFI@tgel was proficient in dissolving established biofilms, with a disruption efficacy reaching 65% and 41% against *P. gingivalis*, 57% and 45% against *PAO1*, and 54% and 40% against *S. mutans* after 24 h and 36 h, respectively. This biofilm eradication efficiency correlates to the capability of OFI@tgel to interfere with the extracellular polymeric substances (EPS), a matrix mainly composed of proteins, glycoproteins, and polysaccharides whereby microorganisms are embedded [48,49]. EPS forms a defense shield for bacteria inside the biofilm and protects them from physical and mechanical stress, the antimicrobial activity of antibiotics, and the host immune system [50]. As expected, a decrease in EPS production—of 45%, 60%, and 54% for *P. gingivalis*, *S. mutans*, and *PAO1*, respectively—was observed in the presence of OFI@tgel with respect to @tgel used as a control (Figure 4B).

Additionally, the biofilm inhibition capability of OFI@tgel was evaluated using the Live/Dead BacLight Bacterial Viability Kit. This assay differentiates between live and dead cells by using propidium iodide, which selectively enters bacteria with damaged membranes and stains them red, while the fluorescent dye Syto9, which stains both live and dead cells, appears green. As shown in Figure 4C, @tgel allowed normal biofilm formation, exhibiting a low live/dead cell ratio, indicating that the bacterial population was in the stationary phase of growth. The addition of OFI@tgel decreased the biofilm viability formed by *P. gingivalis*, *PAO1*, and *S. mutans* with respect to @tgel. These qualitative findings confirmed that the OFI released from the hydrogel effectively inhibited biofilm formation and caused damage to the bacterial cell membranes (Figure 4D).

The cell-to-cell quorum sensing (QS) communication system is the main bacterial process in biofilm development. QS produces and releases into the extracellular matrix chemical signal molecules, called autoinducers, which increase density in both Gram-negative and Gram-positive bacteria (ref). Many studies show that plant extracts inhibit biofilm formation or destroy its structure, likely affecting QS [51]. For instance, research conducted with the OFI extract fruit showed an approximately 80% inhibition effect on the biofilm in *S. mutans* [52]. Evaluation of the expression level of *lasI*, *lasR*, and *rhII*, *rhIR* circuit genes is often used to understand QS-related changes in *PAO1* [53,54]. This system plays a crucial role in the regulation of biofilm formation, virulence, antibiotic resistance, and motility in *PAO1* [54].

To explore the mechanism by which OFI released from the hydrogel inhibits biofilm formation and maturation, the expression levels of several virulence genes were analyzed using qRT-PCR. In particular, *Kgp*, *rgpA*, and *rgpB*, considered important virulence factors and pathogenic markers, were used as targets of *P. gingivalis* QS genes [45,55,56]. The *rhlA* and *rhlB* operons, which are involved in rhamnolipid synthesis, were selected as target quorum sensing (QS) genes in PAO1. For *S. mutans*, the *ComAB* and *ComCDE* operons, which encode the signal transduction system related to the production of virulence factors, were used as targets. [57]. In particular, downregulation of the *comC* and *comD* system could be considered an attractive antibiofilm strategy, since it not only inhibits biofilm formation but also disperses the biofilm [56].

As shown in Figure 4E–G, OFI@tgel significantly (*p* < 0.001) reduced the mRNA levels of all tested genes compared to the hydrogel alone (@tgel). Specifically, when PAO1 was incubated with OFI@tgel, the relative expression levels of the rhlA and rhlB genes decreased 0.48-fold and 0.56-fold, respectively. Similarly, a 0.51-, 0.61- and 0.43-fold reduction in *kgp*, *rgpA*, and *rgpB* relative expression levels was noticed for *P. gingivalis* coltured in the presence of OFI@tgel. Finally, a 0.45- and 0.57-fold decrease in the expression levels of *comC* and *comD* in *S. mutans* was also observed. These data corroborate a study investigating the potentiality of *Opuntia ficus-indica* (L.) Mill peel methanolic extract on the swarming motility and biofilm formation of *PAO1*. The methanol extract significantly decreased the expression level of quorum sensing-related genes (i.e., *lasI*, *lasR*, *rhlI*, and *rhIR*). These results suggest that OFI may inhibit biofilm formation by altering the expression levels of key genes involved in quorum sensing (QS). Therefore, QS system inhibition could be considered an attractive antibiofilm strategy.

### 2.6. OFI@tgel Cellular Internalization and Antioxidant Capacity

In addition to bacterial infections, which can range from superficial paronychia to life-threatening bone infections, periodontitis presents other factors that hinder the healing process, such as reduced collagen synthesis, growth factor production, and impaired migration and proliferation of fibroblasts and odontoblasts. Furthermore, reactive oxygen species (ROS) produced by immune cells exacerbate gingival tissue damage.

**Figure 5:**
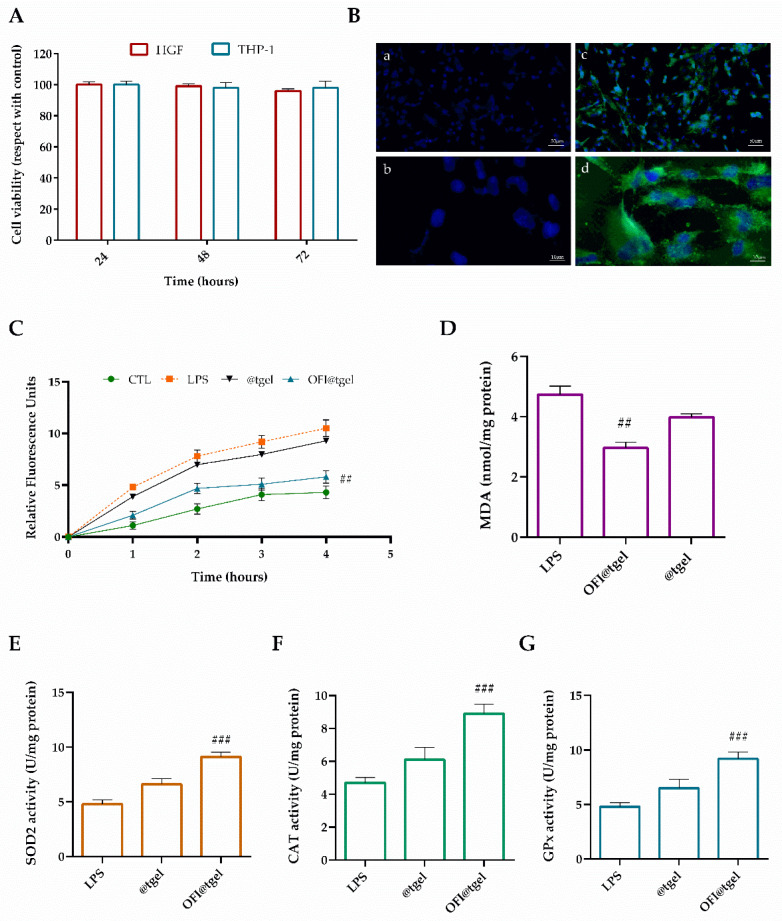
Biocompatibility and antioxidant effect of OFI@tgel on human cells. (**A**) HGF and THP-1 proliferation after 24, 48, and 72 h in the presence of OFI@tgel. (**B**) Fluorescence images of HGF cells treated with OFI@tgel with coumarin-6 for 3 h: (**a**,**c**) 10× magnification; 50 μm scale bar and 40× magnification; 10 μm scale bar (**b**,**d**) (**C**) ROS production was determined by the DCF fluorescence intensity using a microplate reader. (**D**) Malondialdehyde was used as a marker of lipid peroxidation. (**E**) Superoxide dismutase (SOD2), (**F**) catalase (CAT), and (**G**) glutathione peroxidase (GPx) activity was measured using an assay kit. Treatment with LPS of *Porphyromonas gingivalis* (1 μg/mL) is a positive control. Results are expressed as the means of three independent experiments S.D. (*n* = 3). Statistically significant variations: ## *p* < 0.01 and ### *p* < 0.00 versus LPS.

To improve the pro-proliferative and antioxidant therapeutic effects of natural extracts, there has been a growing interest in the development of three-dimensional bioengineered substitutes in the form of films, sponges, microfibers/nanofibers, and hydrogels that can protect and deliver the biomolecules to the target site [58]. Therefore, the biocompatibility and antioxidant activity of OFI@tgel have been evaluated. Firstly, OFI@tgel was found to be biocompatible in both human gingival fibroblasts (HGF) and human monocyte cells (THP-1) at different times, as shown in Figure 5A. Data demonstrated no notable distinction in cell viability after one or three days of co-culture with the OFI@tgel compared to the control group (@tgel), confirming the absence of cytotoxic effects. Subsequently, the ability of the nanoparticles dispersed in the hydrogel to cross the cell membrane was assessed. In particular, OFI-NPs were loaded with a fluorescent molecule, coumarin-6, and then dispersed in the hydrogel. As depicted in Figure 5B, NPs released from the hydrogel showed notable cellular uptake due to the endocytosis-mediated high internalization typical of these nanosystems.

Under healthy periodontal conditions, reactive oxygen species (ROS) help to oxidatively kill pathogens. However, during periodontitis, an imbalance between ROS and antioxidant defense systems acts as intracellular signal transducers, promoting cell death and leading to tissue damage. To mimic periodontal disease, gingival human fibroblasts were cultured in media in the presence of *P. gingivalis* lipopolysaccharide (LPS), an endotoxin portion of the Gram-negative bacterial cell wall that can activate the most abundant pro-inflammatory stimuli.

The effectiveness of OFI@tgel in reducing intracellular ROS generation in human gingival fibroblasts (HGFs) was assessed using the DCFH-DA and MDA assay kits (Figure 5C,D). The DCFH-DA assay measures the ability of redox-active molecules to either inhibit or enhance the oxidation of the probe absorbed by cells into its fluorescent form [59]. Following LPS stimulation, OFI@tgel significantly reduced the formation of oxidants (Figure 5C). Specifically, pre-treatment with OFI@tgel led to a noticeable 55% decrease in ROS production. Excessive lipid peroxidation can damage cell membranes and intracellular organelles, disrupting cell metabolism and leading to broader metabolic disorders affecting periodontal tissues and the entire body [60]. As shown in Figure 5D, LPS treatment caused a significant increase in MDA levels compared to the untreated group, whereas pre-treatment with OFI@tgel resulted in a statistically significant reduction in lipid peroxidation. To counteract ROS-mediated damage, cells produce various antioxidant enzymes, including superoxide dismutases (SOD), catalase (CAT), and glutathione peroxidase (GPx). To determine if the antioxidant effects of OFI@tgel were due not only to its free radical scavenging activity but also to its potential to enhance the endogenous defense system, the activities of SOD2, CAT, and GPx were measured. As illustrated in Figure 5E–G, LPS treatment resulted in a 50% reduction in antioxidant enzyme activity compared to untreated cells. However, pre-treatment of human gingival fibroblasts (HGFs) with OFI@tgel for 24 h led to a significant 45% increase in SOD2 activity relative to LPS-treated cells, indicating effective protection of mitochondria from oxidative damage and restoration of SOD2 activity. Similarly, the activities of CAT and GPx were also restored following OFI@tgel treatment compared to LPS treatment. These results collectively highlight the crucial role of OFI@tgel pre-treatment in suppressing intracellular ROS production, reducing lipid peroxidation, and enhancing antioxidant enzyme activity, thereby mitigating oxidative stress-induced damage in the periodontal in vitro model.

### 2.7. OFI@tgel Modulate the Macrophage Polarization

Macrophages play a role in both the inflammation and restoration of tissue balance caused by periodontitis-related bacterial communities. They achieve this through signaling cascades triggered by pattern recognition receptors in response to different microenvironmental factors [61]. Clinical experiments have shown that the proportion of macrophages increases in the peripheral blood and gingival tissue of patients with chronic periodontitis [62,63].

In periodontitis, macrophages can polarize into different phenotypes, primarily characterized as M1 (classically activated) and M2 (alternatively activated) macrophages. M1 macrophages are pro-inflammatory and are activated in response to microbial products, such as LPS from periodontal pathogens. They secrete tumor necrosis factor-α (TNF-α), interleukin-6(IL-6), interleukin-1β(IL-1β), and other pro-inflammatory factors. Moreover, M1 macrophages produce reactive oxygen species (ROS). Otherwise, M2 macrophages are involved in tissue repair and resolution of inflammation; secrete anti-inflammatory cytokines (e.g., IL-10, IL-4, IL-13), transforming growth factor-β (TGF-β), vascular endothelial growth factor (VEGF), and epidermal growth factor (EGF); and contribute to wound healing, tissue remodeling, and resolution of inflammation in periodontal tissues [61].

**Figure 6:**
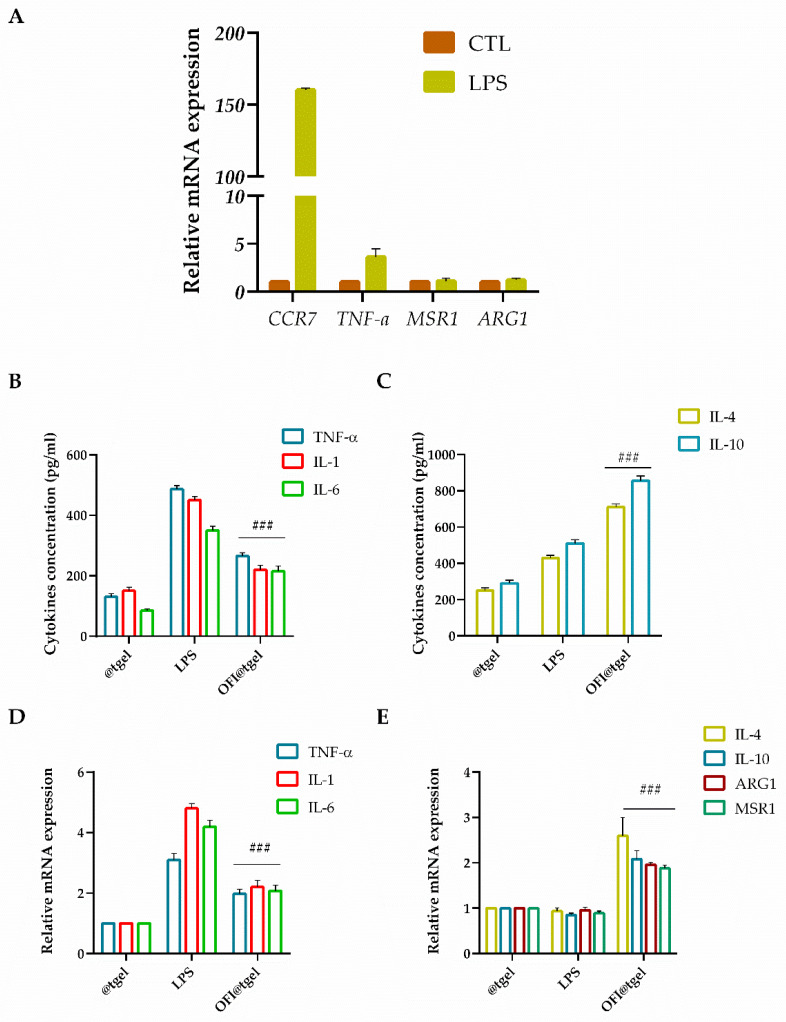
OFI@tgel induction of macrophage M2 polarization. (**A**) mRNA expression of M1 macrophage markers (CTL. CCR7, TNF-α, ARG1, and MSR1) after LPS stimulation (1 μg/mL) for 24 h analyzed using RT-qPCR. Stimulated macrophages were compared with unstimulated cells used as control (@tgel). Data are represented as fold change over actin (2^−ΔΔCt^). Inhibitory effects of OFI on the secretion (**B**) and gene expression (**D**) of inflammatory mediators TNF-α, IL-1 and IL-6 in LPS-stimulated macrophages measured using ELISA assay and RT-qPCR. Cytokine secretion (**C**) and gene expression (**E**) of IL-4, IL-10, ARG1, and MSR1 in LPS-stimulated macrophages measured using ELISA assay and RT-qPCR. THP-1 cells were pre-treated with OFI@tgel for 24 h and then stimulated with LPS for 24 h. PMA-differentiated THP-1 macrophages seeded on the culture plate without LPS stimulation were used as control (@tgel). Results are expressed as the mean of three independent experiments ± S.D. (*n* = 3). Statistically significant variations ### *p* < 0.001 versus gene expression after LPS stimulation.

The balance between M1 and M2 macrophages is crucial in determining the outcome of periodontal disease. Imbalance towards M1 polarization leads to chronic inflammation, tissue destruction, and bone loss. Conversely, a shift towards M2 polarization may promote tissue repair and regeneration. To investigate the effect of OFI@tgel on macrophage polarization, LPS stimulation was employed to simulate the biological response to injury and induce M1 macrophage polarization [64]. As depicted in Figure 6A, LPS stimulation of THP-1 cells significantly increased the expression of chemokine receptor type 7 (CCR7) and elevated TNFα levels (2.6-fold). In contrast, there was no significant expression of macrophage scavenger receptor 1 (MSR1) and arginase 1 (ARG1) genes, which are markers for the M2 macrophage phenotype, indicating that the macrophages were polarized to the M1 phenotype. ELISA results (Figure 6B) revealed that LPS stimulation led to significantly higher production of pro-inflammatory cytokines (TNF-α, IL-1, and IL-6) from macrophages compared to the control group (@tgel). However, in the presence of OFI@tgel, a reduction in these cytokines was observed in the cell culture supernatants (Figure 6B), along with an increase in IL-4 and IL-10 secretion (Figure 6C). Additionally, as shown in Figure 6D, the expression levels of TNF-α, IL-1, and IL-6 were markedly reduced in LPS-stimulated cells treated with OFI@tgel.

The anti-inflammatory factors IL-4 and IL-10 as well as MSR1 and ARG1 were significantly higher in OFI@tgel-treated groups compared to the control group (Figure 6E). As expected, the ability of OFI to modulate the anti-inflammatory response triggers macrophage polarization in the M2 phenotype, creating a microenvironment prone to promoting tissue repair and regeneration.

Understanding macrophage polarization in periodontitis is essential for developing targeted therapeutic strategies to modulate the immune response and promote tissue healing in this prevalent oral disease. Harnessing OFI@tgel therapeutic potential of macrophage polarization pathways offers innovative avenues for developing novel immunomodulatory therapies aimed at restoring immune homeostasis and promoting periodontal tissue regeneration.

Overall, the clinical applications of OFI@tgel primarily focus on its potential as a novel treatment for periodontitis. By providing a sustained and regulated release of bioactive compounds directly within the periodontal pocket, OFI@tgel can more effectively counteract the infection and inflammation associated with periodontitis. Its ability to reduce biofilm formation and modulate the immune response by shifting macrophage polarization from the pro-inflammatory M1 phenotype to the anti-inflammatory M2 phenotype suggests that it could not only halt the progression of periodontitis but also promote tissue healing and regeneration. This makes OFI@tgel a promising candidate for integration into periodontal therapy, offering potentially improved outcomes for patients with chronic periodontal disease. Additionally, its application could be extended to other oral conditions that involve biofilm formation and chronic inflammation, providing a versatile tool for dental practice.

## 3. Materials and Methods

### 3.1. Preparation of OFI Extract

Prickly pears were harvested in September 2022 from farms in Calabria. The samples were transported to the laboratory in refrigerated boxes at 4 °C and processed upon arrival. Prior to peeling, the fruits were thoroughly washed to remove any impurities and spines. The OFI extract was prepared by maceration according to Scarano et al. [16], with some modifications. Specifically, eight grams of the solid mass were treated in the dark with 50 mL of an ethanol/water mixture (EtOH, 8:2 *v*/*v*) under continuous stirring for 8 h. The extracts were then filtered using a Büchner filter (porosity of 4 µm), concentrated with a rotary evaporator, and stored at −20 °C until needed.

### 3.2. Chromatographic Analysis of OFI Extract

For chromatographic analysis and separation, 80 mg of the dried extract was reconstituted in 10 mL of ethanol and sonicated for 60 min at 45 °C. Prior to injection into the mass spectrometry system, the samples were diluted with acetonitrile (1:20 *v*/*v*). Phenolic profiles were determined using a Shimadzu ultra-high-performance liquid chromatograph (Nexera XR) coupled with an MS/MS detector (LCMS 8060, Shimadzu Italy, Milan, Italy), as detailed in the Supplementary Materials. The methods for analyzing total phenol content (TPC) and total flavonoid content (TFC) are also described in the Supplementary Materials.

### 3.3. Synthesis of OFI-Loaded Nanoparticles (OFI-NPs)

Stable and monodisperse OFI-NPs were manufactured employing an automated Dolomite microfluidic system (Dolomite, Royston, UK) equipped with a T-shaped Dolomite glass micromixer chip with H interface connected by 0.25 mm FEP tubing to Mitos Duo XS pumps (10–50 µL/min for the polymer mix and 100–500 µL/min for the extract–TPP solution). Briefly, chitosan solution (0.3% *w*/*v*) in acetic acid (0.1 M) was stirred overnight at room temperature. TPP (0.15 mg/mL) and the dried OFI extract (0.3 mg/mL) were placed in double distilled water under magnetic stirring until complete dissolution. All solutions were filtered using 0.45 µm pore size membrane filters. Different combinations of flow rates and polymer concentrations were explored to evaluate their influence on NP size during the synthetic process (Tables S2 and S3). Based on the set of results obtained, OFI nanoparticles were synthesized at a flow ratio (CS to TPP-OFI extract) of 0.143. OFI-NPs were collected using cooling centrifugation (Frontiers 5718R, OHAUS, Milan, Italy) at 3700 rcf for 45 min at 4 °C, washed with deionized water, and stored at 4 °C until further assays.

### 3.4. Physicochemical Characterization and Biocompatibility Assessment of OFI-NPs

All experimental procedures for evaluating the physicochemical properties and biocompatibility of nanoparticles, including transmission electron microscopy (TEM), Fourier transform infrared spectroscopy (FTIR), nanoparticle tracking analysis (NTA), dynamic light scattering (DLS), stability testing, and encapsulation efficiency, are detailed in the Supplementary Materials.

### 3.5. Preparation and Characterization of OFI-NP Loaded Hydrogel (OFI@tgel)

The OFI@tgel was prepared by uniformly mixing lyophilized OPI-NPs (10 mg) in HA solution for 2 h at 20 °C according to Conte et al. [65]. Characterization of the gelified OFI@tgel was performed using FTIR, water absorption, water retention, in vitro drug release, and biocompatibility. The details are available in the Supplementary Materials.

### 3.6. Antibacterial Studies

#### 3.6.1. Bacterial Strains and Culture Conditions

*Streptococcus mutans* (ATCC 35668), *Porphyromonas gingivalis* (ATCC 33277), and *Pseudomonas aeruginosa* PAO1 (ATCC^®^ BAA-47™) were purchased from the American Type Culture Collection (ATCC, Milan, Italy) and cultured following the ATCC’s guidelines.

#### 3.6.2. Antibacterial Activity of OFI Extract against Pathogenic Bacteria

The antimicrobial activity of the OFI extract released from OFI@tgel was assessed by monitoring the bacterial growth rate of *P. gingivalis*, *S. mutans*, and *PAO1*. For the assay, a bacterial suspension of approximately 1 × 10^5^ CFU/mL was inoculated in 200 µL liquid broth in a 96-well plate, as described by Di Cristo et al. [66]. Then, the plate was incubated at 37 °C and 200 rpm, and at scheduled times (6, 12, and 24 h), the optical density (OD 600 nm) of bacterial suspensions was recorded in a microplate reader (Cytation 3, Bioteck, Arcugnano, Italy). The experiments were performed in triplicate.

#### 3.6.3. Antibiofilm Activity

The capability of the OFI extract to elicit antibiofilm activity was determined as reported by Di Cristo et al. [66] and described in the Supplementary Materials.

#### 3.6.4. Quorum Sensing (QS) Interfering

The ability of OFI@tgel to affect biofilm maturation was assessed using real-time PCR (qRT-PCR) to analyze bacterial mRNA [66]. Total RNA was extracted with TRIzol reagent (Invitrogen, Milan, Italy) and then reverse-transcribed using AMV reverse transcriptase and random hexamers, following the manufacturer’s instructions (Promega, Milan, Italy). Specific primers for sensing genes (*rhlA* and *B* for *PAO1*, *ComC* and *ComD* for *S. mutans*, and *kgp*, *rgpA,* and *B* for *P. ginivalis*) are listed in Table 2.

### 3.7. In Vitro Cell Studies

#### 3.7.1. Human Gingival Fibroblast (HGF) Proliferation

HGFs and THP-1 were cultured in DMEM supplemented with normal DMEM–high glucose (Sigma-Aldrich, St. Louis, MO, USA) and 10% FBS at 37 °C in a humidified atmosphere of 95% air and 5% CO_2_. Images of the cells were captured using a Zeiss microscope (Carl Zeiss, Oberkochen, Germany). For the proliferation assay, HGFs and THP-1 were incubated overnight in low-serum media (0.1% FBS) for 24, 48, and 72 h. The Cell Counting Kit-8 assay (CCK-8, Dojindo Molecular Technologies, Rockville, MD, USA) was carried out as reported by Spagnuolo et al. [67]. Cell absorbance was measured at 450 nm using a microplate reader (Cytation 3; AHSI, Bernareggio, Italy). Cells cultured under standard conditions were used as control. The results were expressed as a percentage relative to the control and calculated using Equation (1):

(1) $$Cytocompatibility\left(\%\right)=\frac{{OD}_{sample}}{{OD}_{control}}\times 100$$

where OD_sample_ is the optical density of the treated cells with NP complexes and OD_control_ is the optical density of the untreated cells.

#### 3.7.2. In Vitro ROS Scavenging Staining

To assess the ability of OFI@tgel to scavenge ROS in vitro, a 500 μL suspension of HGF cells was introduced into 24-well plates at a density of 5 × 10^5^ cells/mL. The plates were then incubated at 37 °C and 5% CO_2_ for 24 h. Next, hydrogel was added to each well through a cellular insert (Corning, Pisa, Italy); in addition, 1 µg/mL of LPS was added. The cells and hydrogels were co-cultured for another 24 h, and a solution of DCFH-DA (10 μM) was introduced to each well, followed by incubation for 4 h. Fluorescence measurements were taken every 5 min for 1 h, with an excitation wavelength of 485 nm and an emission wavelength of 535 nm, using a microplate reader (Cytation 3 Cell Imaging Multi-Mode, ASHI, Milan, Italy). Malondialdehyde (MDA) concentration, an indicator of lipid peroxidation, was assessed using the thiobarbituric acid reactive substances (TBARS) assay. The basal MDA concentration was determined by adding approximately 600 μL of TBARS solution to 50 μg of total protein dissolved in 300 μL of Milli-Q water. The mixture was incubated at 100 °C for 40 min before centrifugation at 14,000 rpm for 2 min. The supernatant was then analyzed using a microplate reader at 532 nm. Total SOD-like, GPx, and CAT activity was measured according to the manufacturer’s protocol described by Valentino et al. [43].

#### 3.7.3. Enzyme-Linked Immunoabsorbent Assay (ELISA)

The levels of the secreted IL-1, IL-6, IL-4, IL-10, and TNF-α proteins were measured in the supernatants of human gingival fibroblasts treated with OFI@tgel and stimulated with LPS (1 µg/mL). Samples and standards (100 µL each) were added to wells pre-coated with antibodies specific for IL-1, IL-6, IL-4, IL-10, and TNF-α. The wells were then incubated for 2 h at 37 °C, following the procedure outlined by Valentino et al. [68].

#### 3.7.4. OFI@tgel Cellular Uptake

Cellular uptake of OFI@tgel was performed using fluorescence microscopy. HGF cells were seeded on a microscope slide at a density of 4.5 × 10^4^ cells in a 24-well plate containing the cell cultured insert. The cells were incubated with OFI@tgel containing fluorescent dye (coumarin) for 3 h at 37 °C. After incubation, the insert was removed and the plate was washed with ice-cold PBS for five times to eliminate non-internalized nanoparticles, and then fixed with paraformaldehyde 3.7% for 15 min. The microscope slides were mounted onto coverslips using ProLong Gold Antifade reagent with DAPI (Thermo Fisher Scientific, Milan, Italy). Images were acquired using the fluorescent microscope AXIO Vert A.1 (Zeiss, Milan, Italy).

#### 3.7.5. Macrophage Polarization to M2 Phenotype

PMA-differentiated THP-1 macrophages were prepared according to the method outlined by Wang et al. [69]. Briefly, cells were seeded in plates or Petri dishes at a density of 5 × 10^5^ cells/mL and incubated for 48 h with 50 ng/mL of phorbol 12-myristate 13-acetate (PMA, Sigma, Milan, Italy). Following PMA differentiation, the cells were washed with PBS and treated with 1 µg/mL of LPS (Sigma) for 4 h to induce the M1 macrophage phenotype [70]. The M1 macrophages were then cultured for 3 days in the presence of OFI@tgel conditioned medium. Macrophage polarization-related genes were analyzed by RT-PCR, as detailed in the Supplementary Materials and in Table S5.

### 3.8. Statistical Analysis

Statistical analysis was conducted using GraphPad Prism software 7.0 (La Jolla, CA, USA). Data are presented as the mean ± standard error of the mean. Statistical differences were assessed using one-way ANOVA, followed by Tukey’s post hoc test for multiple comparisons. Significance levels are indicated as * *p* < 0.05, ** *p* < 0.01, and *** *p* < 0.001.

## 4. Conclusions

In conclusion, the novel OFI-loaded chitosan nanoparticles (OFI-NPs) encapsulated within a thermo-responsive hydrogel (OFI@tgel) represent a significant advancement in periodontal therapy. This innovative approach overcomes the limitations of conventional treatments by providing a sustained and regulated release of bioactive compounds directly within the periodontal pocket. The microfluidic preparation of OFI-NPs ensures precise control over particle size, enhancing their stability and efficacy. The OFI@tgel formulation not only demonstrated effective eradication of key periodontal pathogens, including *S. mutans*, *PAO1*, and *P. gingivalis*, but also interfered with biofilm formation and maturation. Additionally, the hydrogel’s ability to modulate macrophage polarization from the pro-inflammatory M1 phenotype to the anti-inflammatory M2 phenotype underscores its potential in addressing both the microbial and inflammatory aspects of periodontitis. Further clinical studies are needed to validate the translational potential of OFI@tgel in the management of periodontitis and associated pathologies.

## Figures and Tables

**Table 1:** Phenolic acids, flavonoids, and total phenol content in OFI peel extracts. Data are expressed as mean (mg/100 g dry weight) ± standard deviation (SD).

Phenol	Concentration (mg/100 g)
Trans Ferulic Acid	10.10 ± 0.91
Kaempeferol	8.50 ± 0.36
Quercetin	7.90 ± 0.31
Kaempferol-3-O-Glucoside	7.10 ± 0.32
Quercetin-3-O Hexose Deoxyhexose	6.99 ± 0.29
Isorhamnetin	6.83 ± 0.28
Isorhamnetin Rutinoside	6.39 ± 0.25
Ferulic Acid	6.29 ± 0.27
Rutin	6.20 ± 0.21
Caffeic Acid	5.40 ± 0.24
Sinapic Acid	4.83 ± 0.19
Chlorogenic Acid	3.06 ± 0.12
P Coumarci Acid	2.52 ± 0.17
Syringic Acid	1.71 ± 0.14
Kaempferol-3-O-Hexose Deohyhexose	1.31 ± 0.09
Hydroxytyrosol	0.79 ± 0.07
Apigenin	<0.10
Luteoilin	<0.10
Gallocathechin/Epigallocathechin	<0.1

**Table 2:** qRT-PCR primers.

Gene	Forward Primer (5′–3′)	Reverse Primer (5′–3′)
*16SrRNA*	CCTACGGGAGGCAGCAGTAG	CAACAGAGCTTTACGATCCGAAA
*rhlA*	AGCTGGGACGAATACACCA	GACTCCAGGTCGAGGAAATG
*rhlB*	GAGCGACGAACTGACCTACC	GTTGAACTTGGGGTGTACCG
*ComC*	GACTTTAAAGAAATTAAGACTG	AAGCTTGTGTAAAACTTCTGT
*ComD*	CTCTGATTGACCATTCTTCTGG	CATTCTGAGTTTATGCCCCTC
*kgp*	AGGAACGACAAACGCCTCTA	GTCACCAACCAAAGCCAAGA
*rgpA*	CACCGAAGTTCAAACCCCTA	GAGGGTGCAATCAGGACATT
*rgpB*	GCTCGGTCAGGCTCTTTGTA	GGGTAAGCAGATTGGCGATT

## Data Availability

Data is contained within the article and Supplementary Materials.

## Supplementary Materials

The supporting information can be downloaded at https://www.mdpi.com/article/10.3390/ijms25179374/s1.
