# Supplementary material for: Thermo-Responsive Hydrogel Containing Microfluidic Chitosan Nanoparticles Loaded with Opuntia ficus-indica Extract for Periodontitis Treatment
Source: Int J Mol Sci. 2024 Aug 29;25(17):9374. doi: 10.3390/ijms25179374 (PMC11395269; doi:10.3390/ijms25179374)
Supplement: Supplementary file 1 [file ijms-25-09374-s001.zip › ijms-3158061-supplementary.pdf]

Article

Thermo-responsive Hydrogel Containing Microfluidic Chitosan Nanoparticles Loaded with Opuntia ficus-indica Extract for Periodontitis Treatment

Raffaele Conte [1,2,†,*], Anna Valentino [1,2,†], Ilenia De Luca[1], Gemilson Soares Pontes [3,4], Anna Calarco [1,2,*] and Pierfrancesco Cerruti [5]

1 Research Institute on Terrestrial Ecosystems (IRET)-CNR, Via Pietro Castellino 111, 80131 Naples, Italy; raffaele-conte@cnr.it R.C.); anna.valentino@cnr.it (A.V.); ilenia.deluca@cnr.it (I.DL.); anna.calarco@cnr.it (A.C.)
2 National Biodiversity Future Center (NBFC), 90133 Palermo, Italy
3 Laboratory of Virology and Immunology, National Institute of Amazonian Research (INPA), Manaus, AM, Brazil; gemilson.pontes@inpa.gov.br. (G.P.)
4 Post-Graduate Program in Basic and Applied Immunology, Institute of Biological Science, Federal University of Amazonas, Manaus, AM, Brazil
5 Institute for Polymers, Composites, and Biomaterials (IPCB-CNR), Via Campi Flegrei 34, 80078 Pozzuoli (NA), Italy. pierfrancesco.cerruti@cnr.it; (P.C.)
† These authors contributed equally to this work
* Correspondence: raffaele-conte@cnr.it; anna.calarco@cnr.it

1. RESULTS

1.1 Identification of (poly)phenolic compounds by mass spectroscopy

For chromatographic analysis and separation, 80 mg of dried extract was resuspended in 10 ml of ethanol. The mixture was sonicated for 60 min at 45 °C. For efficient injection into the bulk system, further dilution of the sample with acetonitrile (1:20 v/v) was required. A Shimadzu Ultra-High-Performance Liquid Chromatograph (Nexera XR) combined with an MS/MS detector (LCMS 8060, Shimadzu Italy, Milan, Italy) was used to determine the phenolic profile of the sample. Electrospray ionization in negative mode was employed for detection. Chromatographic separation was obtained in isocratic conditions using acetonitrile:water + 0.01 % formic acid (5:95, v/v) as mobile phase and Kinetex 2.6 µm C18 100 Å, LC Column 100 x 4.6 mm (Phenomenex Inc., U.S.A) as stationary phase. Mass conditions were set as follows: nebulizing gas flow: 3 L/min, heating gas flow: 10 L/min, interface temperature: 300 °C, DL temperature: 250 °C, heat block temperature: 400 °C, and drying gas flow: 10 L/min. The polyphenols analyzed in the sample are included in Table S1.

Table S1. Screening method for polyphenols.

Name	m/z (ESI-)
Syringic Acid	198,90
Gallic Acid	168,90
Quercetin	301,00
P Coumarci Acid	162,90
Oleochantal	303,20
Hydroxytyrosol	153,05
Trans Ferulic Acid	193,00
Oleuropein	539,00
Hesperetin	301,30
Trimethoxyflavone	312,00
Arbutin	271,20
Rosmarinic Acid	359,00
Ursolic Acid	455,00
Apigenin	269,00
Amentoflavone	537,10
Luteoilin	284,90
Quercetin-3-O-Glucoside	463,10
Quercetin-3-O- Glucuronic Acid	477,00
Kaempferol-3-O-Glucose	609,10
Quercetin-3-O.Hexose Deoxyhexose	609,10
Isorhamnetin- 3-O Rutinoside	623,10
Isorhamnetin-7-O- Pentose	447,10
Luteoilin 7-O-Glucoside	447,10
Kaempferol-3-O-Glucuronic Acid	461,10
Kaempferol-3-O-Pentose	417,10
Kaempferol-3-O-Hexose Deohyhexose	593,10
Tyrosol	153,40

Protocathecoic Acid	153,00
Vanillic Acid	167,00
Syringic Acid	197,00
P-Hydroxybenzoic\Salicilic Acid	137,00
Gentisic Acid	153,00
Caffeic Acid	179,00
Sinapic Acid	223,00
Ferulic Acid	193,00
Trans-Cinnamic Acid	147,00
Chlorogenic Acid	353,00
Cathechin\Epicathechin	289,00
Gallocathechin\Epigallocathechin Gallate	457,00
Gallocathechin\Epigallocathechin	305,00
Cathechin Gallate	441,00
Procianidin	577,00
Myricetin	317,00
Kaempeferol	285,00
Rutin	609,00
Narigin	579,00

1.2 OFI-NPs Synthesis

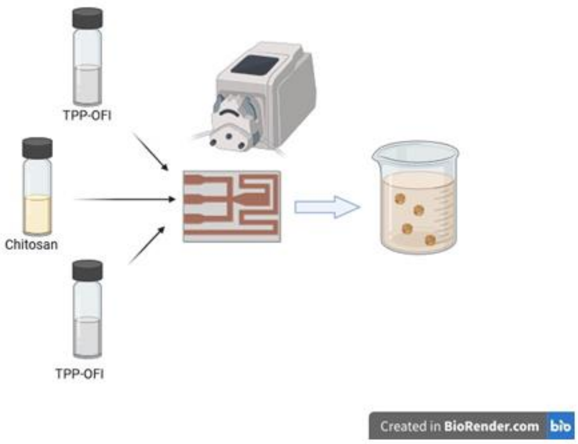

Figure S1. Schematic representation of OFI-NPs synthesis. OFI-NPs were synthesized through a micromixer chip that allowed the rapid mix of Chitosan in 0.3 % w/v in acetic acid with a solution of TPP (0.15 mg/mL) containing OFI 0.6 mg\ml in water. Cross linking agent flowed in the lateral channels, whereas the polymeric solution in the middle channel. The final product is represented by Chitosan NPs containing OFI extract.

OFI-NPs were synthesized using the automated Dolomite microfluidic system (Dolomite, Royston, UK), equipped with a T-shaped microfluidic chip (figure S1), following the procedure detailed in the manuscript. The manufacturing parameters—including core (middle) flow speed, sheath (side) flow speed, flow rate ratio (FRR), chitosan concentration, and sodium tripolyphosphate (TPP) concentration—were systematically optimized to achieve monodispersity of the nanoparticles. Initially, the optimization focused on the flow rate ratio (FRR) using fixed concentrations of chitosan (0.5% w/v) and TPP (0.15% w/v), as shown in Table S2. Subsequently, both the polymer (chitosan) and crosslinker (TPP)

concentrations, along with their respective flow rates, were further optimized. These optimizations are summarized in Table S3.

Table S2. Optimization of flow rate ratio.

Middle stream (µl/min)	Side stream (µl/min)	Flow rate ratio	Nanoparticle size (nm)
10	100	0.1	139 ± 13
20	100	0.2	216 ± 11
30	100	0.3	315 ± 18
40	100	0.4	387 ± 19
50	100	0.5	471 ± 24
10	200	0.05	287 ± 17
20	200	0.1	121 ± 19
30	200	0.15	106 ± 12
40	200	0.2	222 ± 18
50	200	0.25	254 ± 19
10	350	0.028	334 ± 22
20	350	0.057	261 ± 16
30	350	0.086	192 ± 13
40	350	0.114	109 ± 12
**50**	**350**	**0.143**	**92 ± 13**

Table S3. CS-NPs synthesis optimization.

Chitosan concentration (% w/v)	TPP concentration (mg/ml)	Middle stream (µl/min)	Side stream (µl/min)	Nanoparticle size (nm)	Polidispersivity (PDI)
0.10	0.15	25	175	116 ± 20	0.298
0.20	0.15	25	175	108 ± 19	0.307
0.30	0.15	25	175	93 ± 12	0.188
0.40	0.15	25	175	124 ± 22	0.209
0.50	0.15	25	175	153 ± 27	0.234
0.10	0.15	50	350	120 ± 15	0.121
0.20	0.15	50	350	115 ± 13	0.098
**0.30**	**0.15**	**50**	**350**	**90 ± 11**	**0.046**
0.40	0.15	50	350	131 ± 16	0.087
0.50	0.15	50	350	169 ± 21	0.103
0.10	0.15	30	210	119 ± 17	0.138
0.20	0.15	30	210	128 ± 19	0.116
0.30	0.15	30	210	92 ± 13	0.088
0.40	0.15	30	210	124 ± 21	0.109
0.50	0.15	30	210	133 ± 23	0.116
0.10	0.15	15	105	100 ± 19	0.456
0.20	0.15	15	105	93 ± 25	0.357
0.30	0.15	15	105	90 ± 20	0.241
0.40	0.15	15	105	119 ± 15	0.451
0.50	0.15	15	105	130 ± 27	0.484

The optimized conditions (in bold) were utilized to synthesize nanoparticles encapsulating *Opuntia Ficus Indica* extract (OFI). The incorporation of different amounts of dried OFI extract significantly influenced the nanoparticles' polydispersity index (PDI) and encapsulation efficiency. These impacts are detailed in

Table S4, demonstrating how varying the OFI extract concentration affects the uniformity and encapsulation properties of the nanoparticles.

Table S4. OFI-NPs synthesis optimization.

Chitosan concentration (% w/v)	TPP concentration (mg/ml)	Middle stream (µl/min)	Side stream (µl/min)	OFI extract concentration (mg/ml)	Nanoparticle size (nm)	Polidispersivity (PDI)	Encapsulation efficency (EE%)	Z potential (mV)
0.30	0.15	50	350	0.1	95 ± 10	0.063	20	32.4 ± 0.5
0.30	0.15	50	350	0.2	98 ± 13	0.065	34	31.3 ± 0.7
0.30	0.15	50	350	0.4	96 ± 12	0.066	48	30.4 ± 0.6
0.30	0.15	50	350	0.5	104 ± 16	0.219	69	31.2 ± 0.7
**0.30**	**0.15**	**50**	**350**	**0.6**	**100 ± 13**	**0.068**	**75**	**30.9 ± 0.5**
0.30	0.15	50	350	0.8	135 ± 21	0.238	67	27.1 ± 1.1
0.30	0.15	50	350	1	166 ± 23	0.334	66	19.9 ± 1.3
0.30	0.15	50	350	1.2	223 ± 27	0.381	70	12.3 ± 1.63

1.3 Antioxidant ability of OFI@tgel

To establish the antioxidant ability of OFI released from hydrogel, two complementary cell-free tests were used (DPPH and FRAP). The DPPH assay measures the ability of a bioactive molecule to inhibit lipid oxidation, while the FRAP assay is based on electron-transfer reactions where a molecule is able to reduce Fe3+-tripyridyltriazine to the colored Fe2+-tripyridyltriazine.

The results confirmed the ability of released OFI to react with free radicals or terminate chain reactions, with a more noticeable radical scavenging efficiency of the OFI@tgel with respect to OFI alone (Figure S2). While the scavenging efficiency of pure OFI extract decreases over time, the presence of @tgel maintains the OFI antioxidant activity constant for the experiment's duration.

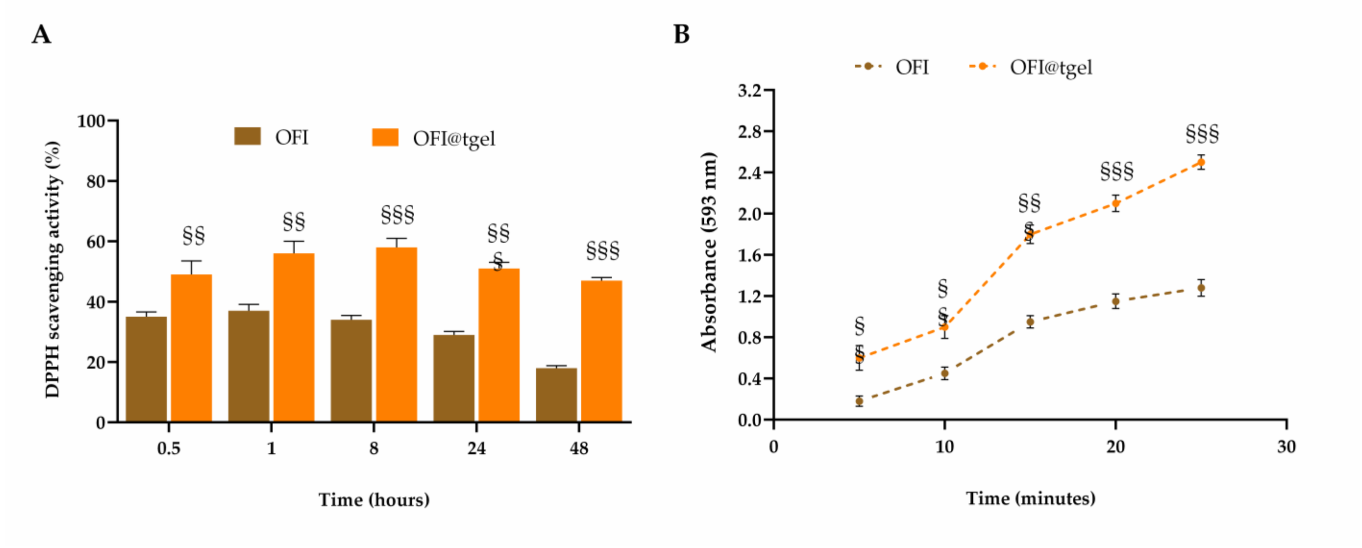

Figure S2: OFI@tgel antioxidant activity. DPPH scavenging activity (**A**), and ferric reducing power (FRAP assay) (**B**) of OFI, and OFI@tgel. For each sample, six different experiments were carried out, and the results are expressed as the means of the values obtained (mean ± SD). Statistically significant variations: §§ $p < 0.01$ and §§§ $p<0.001$ versus OFI.

2. MATERIALS AND METHODS

2.1 Materials.

Chitosan (medium molecular weight, 50,000–190,000 Da, 75–85% deacetylated, viscosity < 200 mPa.s, 1% in acetic acid), Acetic acid (Glacial acetic acid, CAS 64-19-7), sodium tripolyphosphate (TPP, technical grade), Coumarin and Ultrapure HA in the form of sodium hyaluronate medium molecular weight were purchased from Sigma-Aldrich (Milan, Italy) and used as received. Micromixer chip #3200401 was purchased from Dolomite (PT). Phosphate-buffered saline (PBS) Water obtained from a Milli-Q® Direct Water Purification System was used in all the experiments. All other reagents used in the experiment were of analytical grade and, when not indicated, were purchased from Sigma-Aldrich (Milan, Italy).

2.2 Total Phenolic Content

Total Phenolic content (TPC) was obtained by the Folin-Ciocalteu method as already reported (Guevara-Figueroa et al., 2010). The ethanolic extract (20 µL) was added to 1.58 mL of MilliQ water and mixed. Then 300 µL of 20% Na_2CO_3 and 100 µL of Folin-Ciocalteu phenol reagent were added and the mixture was allowed to stand for 2 h at room temperature. Absorption of the solution was read at 765 nm in a UV-Vis spectrophotometer (Varian Cary, Palo Alto, CA, USA). Total phenolic content was expressed as µmol of gallic acid equivalents/g of sample. All determinations were performed in triplicate.

2.3 Total flavonoid content

Flavonoid quantification was performed following the reported method (Guevara-Figueroa et al., 2010), with some modifications. Briefly, 15 µL of extract were added to 735 µL of ultra-pure water and mixed with 750 µL of 2% AlCl3 solution. After 10 min, the mixture absorbance was read at 367 nm in a UV-Vis Spectrophotometer (Varian Cary). Quercetin was used as a reference standard and results were expressed as µmol quercetin equivalents/g of sample. All determinations were performed in triplicate.

2.4 OFI-NPs Characterization

Particle size (hydrodynamic diameter), polydispersity index (PDI), and zeta potential measurements were carried out on freshly prepared samples. The reported data are an average value of three measurements of the same sample.

2.4.1 Dynamic light scattering (DLS)

The size distribution of OFI-NPs was analyzed by DLS (Zetasizer Ultra, Malvern Panalytical, Amesbury, UK). The DLS technique analyzes the velocity distribution of particle motion caused by Brownian motion by measuring dynamic fluctuations in the intensity of scattered light. The hydrodynamic radius of the particle, or diameter considered, was calculated with the Stokes–Einstein equation. Ten microliters of purified OFI-NPs were diluted in 990 µL of filtered PBS and vortexed.

2.4.2 Nanoparticles Tracking Analysis (NTA)

NTA from Malvern (Malvern Panalytical Ltd., Malvern, Worcestershire, UK) was used for the measurement of size distribution and concentration of OFI-NPs samples in the liquid suspension. Briefly, samples were diluted with 0.2 µm-filtered PBS to obtain a recommended measurement concentration between ~1.1 $*10^9$ particles/mL. Five videos of typical 60 s duration were taken. Data were analyzed using NanoSight NTA software version 3.2, which was optimized to first identify and then track each particle on a frame-by-frame basis with a 488 nm laser. The temperature was maintained at 25 °C. Filtered PBS (blank) was run as a negative control.

2.4.3 Transmission Electron Microscopy (TEM)

After isolation, OFI-NPs was resuspended in PBS and properly diluted, and then incubated for 5 min onto carbon-coated copper grids, 200 mesh at room temperature. Once absorbed on the grids, OFI-NPs was fixed with 2% glutaraldehyde in PBS for 10 min and then washed three times in Milli-Q water. Negative staining was performed with 2% phosphotungstic acid; finally, the grids were air-dried and observed by an FEI Tecnai G2 Spirit TWIN 120 kV with emission source LaB6 and mounting FEI Eagle 4k CCD camera (on the bottom) and Olympus SIS.

2.5 Stability test and encapsulation efficiency

Stability tests were performed once a week for up to three months. The samples were split into three batches and stored at 25°C. Each week, the particles' diameter, PDI, and ζ potential were assessed by DLS.
The amount of drug entrapped in NPs was determined indirectly by quantifying the amount of free trans ferulic acid (one of the main components of OFI extract) in the supernatant using its characteristic m\z of 193 (ESI-). The encapsulation efficiencies of a series of OFI-loaded nanoparticles were expressed based on the following equation:

$$\boldsymbol{Encapsulation\ Efficiency\ (EE\%)} = \frac{\textbf{Total amount of ferulic acid loaded} - \textbf{Free ferulic acid in supernatan}}{\textbf{Total amount of ferulic acid loaded}} \times \mathbf{100}$$

2.6 OFI@tgel Characterization

The water absorption capacity of the hydrogel was assessed by the weighting method. Hydrogel was lyophilized and weighted (M1). Then, it was immersed in simulated salivary fluid (SSF) at pH 6.5 and constant temperature (35°C). Periodically, the hydrogel was taken out, weighed and measurements were recorded as Mt. The absorption ratio was calculated according to the following equation:

$$\text{absorption ratio (\%)} = \frac{\text{Mt-M1}}{\text{M1}} \times 100\%$$

For water retention, hydrogel was swollen to saturation at 35°C and weighed (Ms). Then, the weight of the hydrogel was periodically measured and recorded (Mt). The water retention rate was calculated according to the following equation:

$$\text{Water retention(\%)} = \frac{\text{Mt}}{\text{Ms}} \times 100\%$$

2.7 Antibiofilm activity

The capability of OFI extract to elicit antibiofilm activity was determined as reported by Di Cristo et al. Briefly, a similar amount of released OFI extract was placed in a 48-well polystyrene plate and covered by 800 µL of liquid medium broth containing P. gingivalis, S. mutans, and PAO1 (1 × 107 CFU/mL). OFI extract incubated in liquid medium broth was used as negative control, while 800 µL of PAO1, S. mutans and P. gingivalis (1 × 107 CFU/mL) were used as positive controls. Cultures were incubated statically at 37 °C in a humid atmosphere until a mature biofilm was obtained. After 6, 12, and 24 h the surface-adhered biofilm was gently washed with sterile PBS, air-dried for 30 min, and stained with 0.1% w/v crystal violet (CV). CV assay was used to determine biofilm formation, as previously described (Di Salle et al.). Briefly, at the established time (6, 12, and 24 h), each well was gently washed with sterile PBS to remove non-attached bacteria, air-dried for 30 minutes, and stained with 0.1% w/v CV. The stained biofilm was solubilized in 96% ethanol and quantified by measuring the optical density (OD) at 570 nm using a microplate reader (Cytation 3, Bioteck). Measurements were carried out in triplicate.

2.8 ROS scavenging ability

The antioxidant capacity of OFI and OFI@tgel was determined using DPPH and FRAP assays (Figure S2). In DPPH assay, the scavenging activity of OFI, OFI-NPs, and OFI@tgel was spectrophotometrically determined at 517 nm using a microplate reader (Cytation 3, BioTek) following the formula:

$$\textbf{Scavenging activity}\ (\%) = \frac{A_0 - A_1}{A_0} \times \mathbf{100}\%$$

where A_1 is the absorbance of the experimental group and A_0 is the absorbance of the DPPH solution.

The ferric-reducing antioxidant power (FRAP) assay deals with the reducing ability of antioxidants, which involves reduction in Fe3+-2,4,6-tripyridyl-s-triazine (TPTZ) complex while taking absorbance at 593 nm. The FRAP reagent is prepared by taking acetate buffer 3.6 pH, 10 mmol of TPTZ solution in 40 mmol of hydrochloric acid (HCl), and 20 mmol solution of iron (III) chloride in 10:1:1 (v/v) ratio. Then, 5 µL sample (0.5–2 mg/mL) gets diluted with distilled water (20 µL) and added to the FRAP reagent (150 µL). A microplate spectrophotometer reader (Cytation 3 BioTek) measures the absorbance after 8 min at 593 nm.

2.9 Real-Time Quantitative PCR (RT-qPCR)

Anti-inflammatory activity was evaluated by Real-Time Quantitative PCR (RT-qPCR) according to the manufacturer's protocols. For RT-qPCR, total RNA was extracted from cells with and without OFI@tgel stimulated with LPS (1 µg/mL) through TriFast (EuroClone, Milan, Italy), and cDNA was synthesized using Wonder RT cDNA synthesis Kit (EuroClone). Then, gene expressions of TNF-α, IL-1, IL-4, IL-6, IL-10, ARG1, and MSG1 were evaluated by 7900 HT fast Real-Time PCR System, (Applied Biosystem, Foster City, CA, USA) with SYBR Green PCR Master mix (EuroClone). Gene expression was quantified by the $2^{-\Delta\Delta Ct}$ method and normalized against β-actin used as the internal reference gene. Results were expressed as fold changes versus control. Primers used for RT-qPCR are reported in Table S5.

Gene	Accession number	Forward (5′-3′)	Reverse (5′-3′)
**IL-1**	NM_000576.3	GGAGAATGACCTGAGCACCT	TGATCGTACAGGTGCATCGT
**IL-4**	NM_000589.4	GCAGTTCTACAGCCACCATG	GTCGAGCCGTTTCAGGAATC
**IL-6**	NM_000600.5	CGCCTTCGGTCCAGTTGCC	GCCAGTGCCTCTTTGCTGCTTT
**IL-10**	NM_000572.3	GGGGCTTCCTAACTGCTACA	AGTGGTTGGGGAATGAGGTT
**TNF-α**	NM_000594.4	AACATCCAACCTTCCCAAACGC	TGGTCTCCAGATTCCAGATGTCAGG
**ARG1**	NM_000045.4	TGGAAGTGAACCCATCCCTG	AGGCTTGTGATTACCCTCCC

**MSR1**	NM_138715.3	GCTTTGCTTCCTCCGAATCC	ACTGCAAACACGAGGAGGTA
**ACTB**	NM_001101.5	ACTCTTCCAGCCTTCCTTCC	CGTACAGGTCTTTGCGGATG

Table S5: Primers used for qRT-PCR.
